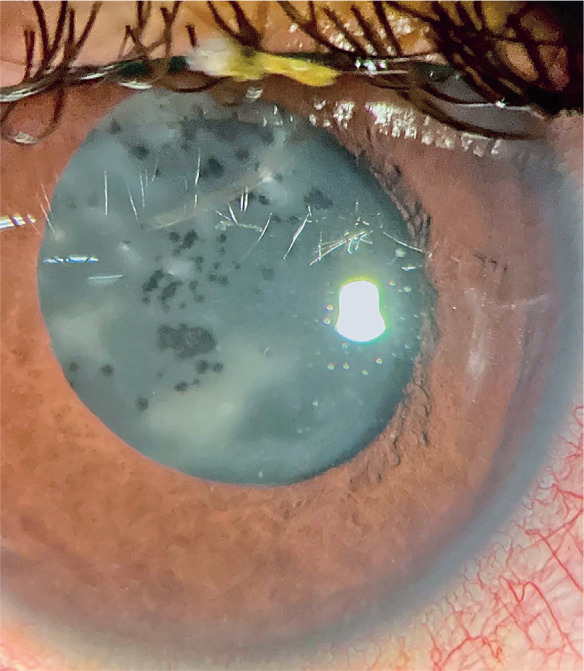# Intraocular lens opacification

**DOI:** 10.5935/0004-2749.2024-0403

**Published:** 2025-02-11

**Authors:** Pedro Henrique Oliveira Ribeiro, Nicole Bulgarão Maricondi de Almeida, Newton Kara-Júnior

**Affiliations:** 1 Ophthalmology department, Hospital das Clínicas, Universidade de São Paulo, São Paulo, SP, Brazil

Although intraocular lens opacification is uncommon, multiple different patterns of opacities have been des-cribed according to the intraocular lens material. A well-known complication of silicone lenses is the adherence of silicone droplets on the lens surface after silicone oil injection at the time of retinal surgery. These droplets are nearly impossible to displace (Figure 1). Furthermore, these droplets hamper the patient’s vision as well as the physician’s view of the posterior pole. Thus, the compromised intraocular lens will require an exchange^(1)^.

**Figure:**